# Application of a pulmonary nodule detection program using AI technology to ultra-low-dose CT: differences in detection ability among various image reconstruction methods

**DOI:** 10.1007/s11604-025-01781-x

**Published:** 2025-05-09

**Authors:** Nanae Tsuchiya, Shifumi Kobayashi, Ryo Nakachi, Yukari Tomori, Akira Yogi, Gyo Iida, Junji Ito, Akihiro Nishie

**Affiliations:** 1 Department of Radiology, Graduate School of Medical Science, University of the Ryukyus, 1076 Kiyuna, Ginowan-shi, Okinawa 901-2720 Japan; 2 Division of Radiology, Department of Medical Technology, University of the Ryukyus Hospital, Okinawa, Japan

**Keywords:** Ultra-low-dose, Computed tomography, Lung nodule, Artificial intelligence, Deep learning reconstruction, Computer-aided detection

## Abstract

**Purpose:**

This study aimed to investigate the performance of an artificial intelligence (AI)-based lung nodule detection program in ultra-low-dose CT (ULDCT) imaging, with a focus on the influence of various image reconstruction methods on detection accuracy.

**Methods:**

A chest phantom embedded with artificial lung nodules (solid and ground-glass nodules [GGNs]; diameters: 12 mm, 8 mm, 5 mm, and 3 mm) was scanned using six combinations of tube currents (160 mA, 80 mA, and 10 mA) and voltages (120 kV and 80 kV) on a Canon Aquilion One CT scanner. Images were reconstructed using filtered back projection (FBP), hybrid iterative reconstruction (HIR), model-based iterative reconstruction (MBIR), and deep learning reconstruction (DLR). Nodule detection was performed using an AI-based lung nodule detection program, and performance metrics were analyzed across different reconstruction methods and radiation dose protocols.

**Results:**

At the lowest dose protocol (80 kV, 10 mA), FBP showed a 0% detection rate for all nodule sizes. HIR and DLR consistently achieved 100% detection rates for solid nodules ≥ 5 mm and GGNs ≥ 8 mm. No method detected 3 mm GGNs under any protocol. DLR demonstrated the highest detection rates, even under ultra-low-dose settings, while maintaining high image quality.

**Conclusion:**

AI-based lung nodule detection in ULDCT is strongly dependent on the choice of image reconstruction method.

## Introduction

Recent advancements in artificial intelligence (AI) have significantly impacted various domains of healthcare, including radiology. One of the pivotal innovations in this field is deep learning reconstruction (DLR), which leverages convolutional neural networks to reduce noise and artifacts in medical imaging [1–5]. By improving image quality, DLR has enabled accurate evaluation of pulmonary nodules, even in ultra-low-dose (ULD) computed tomography (CT) scans, a critical advancement given the ongoing push to minimize radiation exposure in lung cancer screening. The average effective radiation dose for lung cancer screening using low-dose CT (LDCT) is approximately 1.5–2.0 mSv, which is about one-quarter to one-fifth of that of conventional chest CT. However, a technique known as ultra-low-dose CT (ULDCT), which further reduces the effective radiation dose to 0.13–0.44 mSv, has been reported [6]. Concurrently, AI technologies have been integrated into computer-aided detection (CAD) systems to assist radiologists in identifying pulmonary nodules more efficiently and accurately [5, 7–9]. These systems have proven effective in mitigating errors due to human oversight and enhancing the efficiency of interpretation of the increasing volume of CT imaging data in clinical practice.

Despite these advancements, the effectiveness of CAD systems in detecting pulmonary nodules in DLR-reconstructed ULD CT images has not been extensively validated. Previous studies have indicated the superiority of DLR over conventional image reconstruction methods such as filtered back projection (FBP) and iterative reconstruction, with DLR demonstrating improved nodule detection rates and measurement accuracy under low-dose settings [1–5]. However, there remains a gap in understanding whether this superiority translates to enhanced nodule detection performance when integrated with AI-based CAD systems.

In this study we hypothesized that DLR-reconstructed images would lead to improved pulmonary nodule detection by CAD systems compared to traditional reconstruction methods. To test this hypothesis, we employed a chest phantom embedded with artificial lung nodules scanned under ULD CT protocols. We then evaluated the performance of a CAD system in detecting these nodules across different reconstruction methodologies. This investigation aims to elucidate the role of advanced reconstruction methods in enhancing AI-assisted lung cancer screening accuracy and should ultimately contribute to safer, more reliable early diagnosis protocols.

## Materials and methods

### Phantoms

**Fig. 1:**
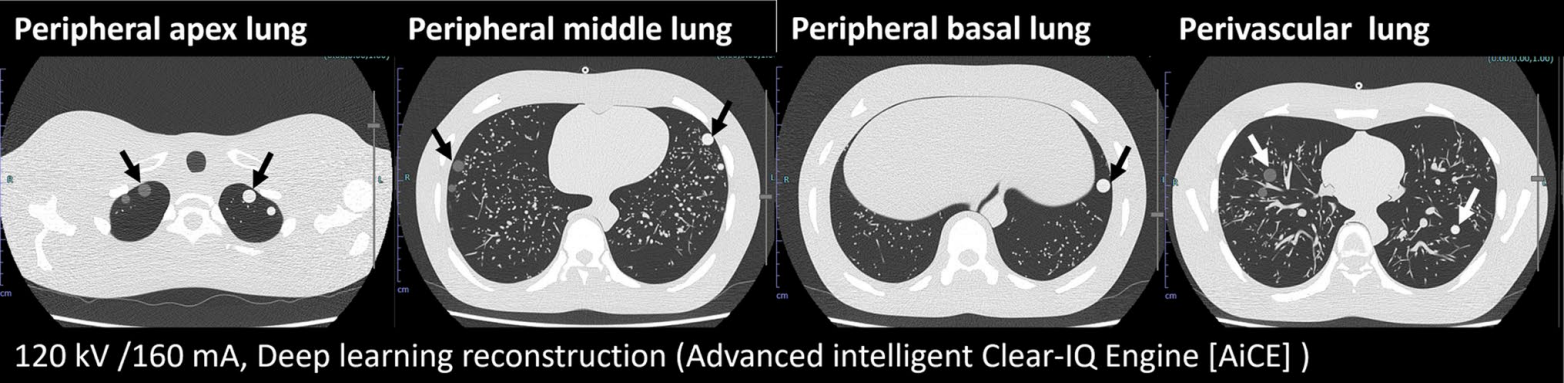
The artificial lung nodules were placed in four locations on the chest phantom: the peripheral lung apex level, the peripheral middle lung level, the peripheral lung basal level, and the perivascular lung

A chest X-ray phantom that anatomically reproduced the mediastinum and lung field structure was used (Chest Phantom N-1 LUNGMAN; Kyoto Kagaku, Kyoto, Japan). The lung parenchyma of the phantom is composed of a combination of urethane foam and styrene resin with a CT value of approximately − 900 HU. Four different sizes (12 mm, 8 mm, 5 mm, and 3 mm diameter) of solid (100 HU) and ground-glass (−630 HU) spherical artificial lung nodules were placed in the chest X-ray phantom. A total of eight artificial nodules were placed at each of the peripheral lung apex level, the peripheral middle lung level, the peripheral lung basal level, and the perivascular lung of the phantom (Fig. 1).

### CT scan and image reconstruction

**Fig. 2:**
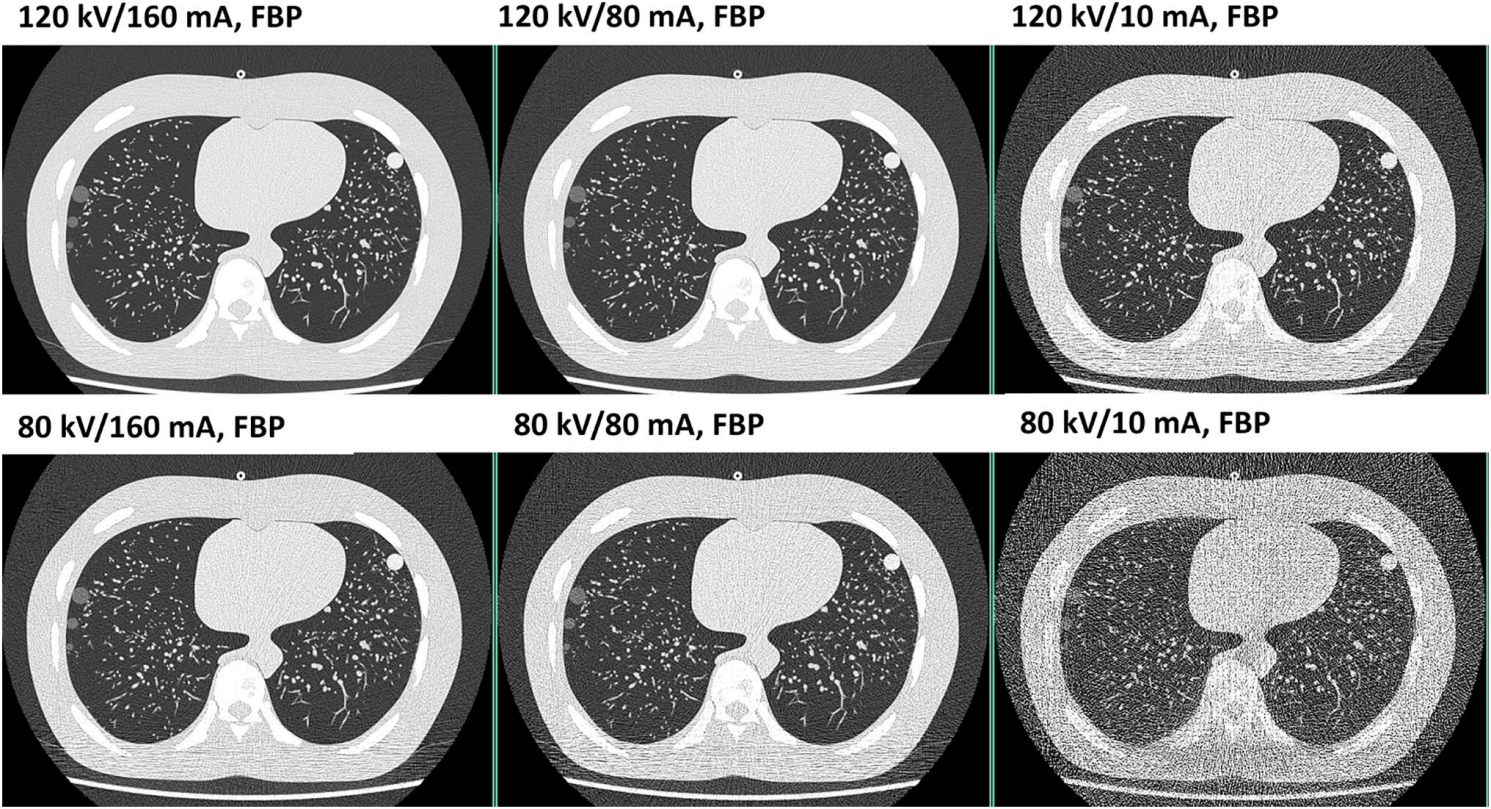
A chest phantom containing artificial lung nodules was scanned under various dose settings. The CT protocol included six distinct configurations, combining tube currents of 160, 80, and 10 mA with tube voltages of 120 and 80 kV. The images were reconstructed by filtered back projection (FBP)

**Fig. 3:**
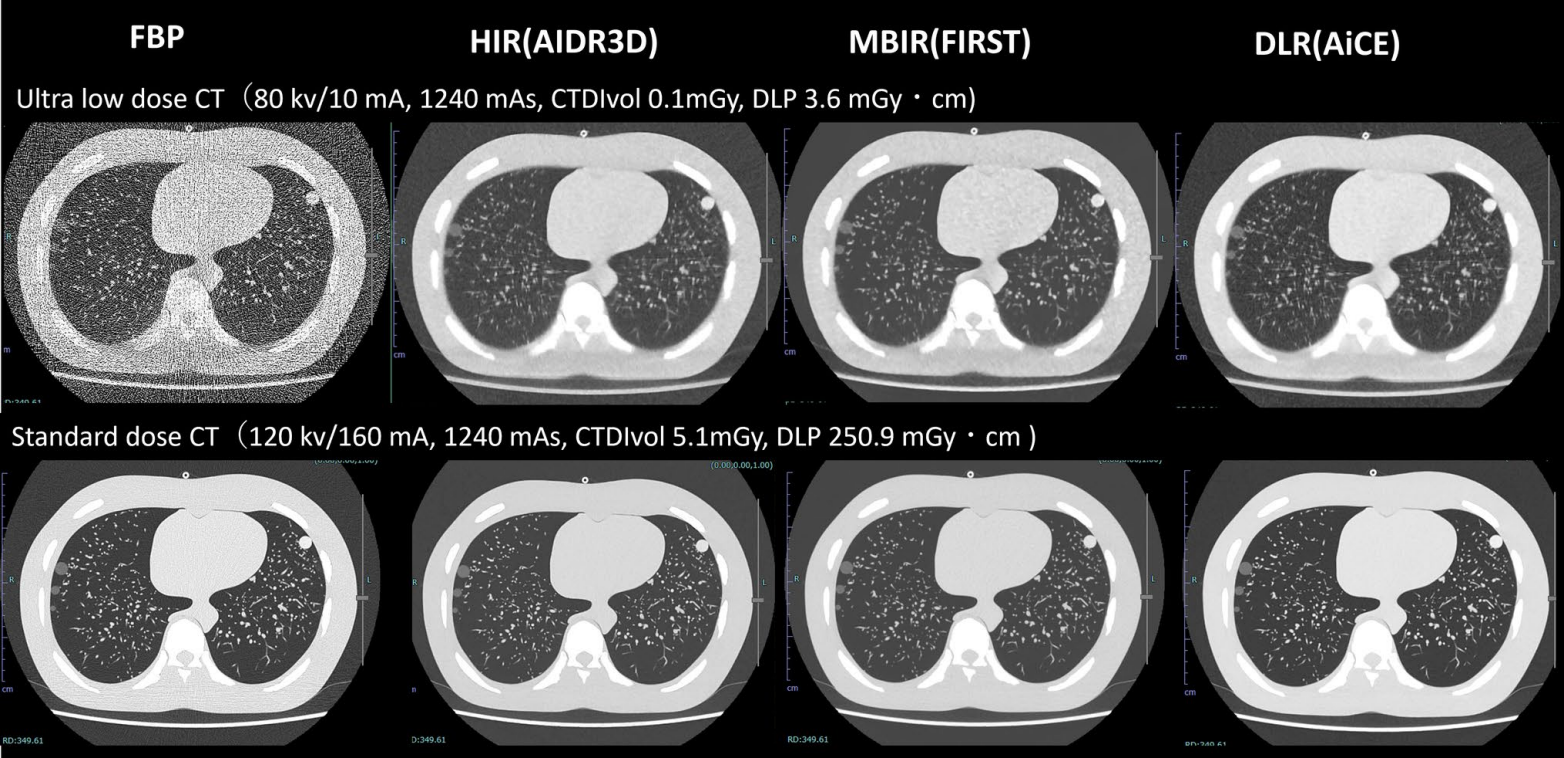
The upper images were obtained using ultra-low-dose CT, while the lower images were acquired by standard dose CT. The collected images were generated using four image reconstruction methods: FBP (filtered back projection; HIR (hybrid iterative reconstruction); MBIR (model-based iterative reconstruction); and DLR (deep learning reconstruction). The image reconstruction methods made a greater contribution to noise reduction in the ultra-low-dose CT

The chest phantom with artificial lung nodules was scanned on a 320-row detector CT system (Aquilion ONE/PRISM Edition; Canon Medical Systems, Otawara, Japan). The following scanning parameters were used: X-ray tube voltage, 120 kV and 80 kV; detector configuration, 0.5 mm × 80 rows; beam pitch, 0.8; gantry rotation time, 0.5 s; and X-ray tube current, 160, 80, and 10 mA. Thus, six different combinations of tube voltage and tube current were acquired three times consecutively (Fig. 2). Each set of raw data was reconstructed using FBP (filtered back projection), HIR (hybrid iterative reconstruction; herein using the adaptive iterative dose reduction 3D [AIDR 3D] standard FC52 algorithm), MBIR (model-based iterative reconstruction; using the forward projected model-based iterative reconstruction solution [FIRST] LUNG algorithm), and DLR (deep learning reconstruction; using the advanced intelligent clear-IQ engine [AiCE] LUNG algorithm) (Fig. 3). The lung kernel reconstruction was adopted for the detection of lung nodules. The reconstruction time of each reconstruction methods was recorded. Radiation doses of each scan were recorded in terms of the volume CT dose index (CTDIvol [mGy]) and the total dose length product (DLP [mGy cm]). CTDIvol and DLP values were obtained from the radiation dose sheet generated by the CT scanner.

### Image quality

To compare the quantitative image quality among FBP, HIR, MBIR, and DLR at each dose setting, the mean CT values and standard deviation (SD) of CT values for each 12-mm-diameter artificial nodule (solid and GGN) and lung parenchyma at the same slice level were measured by circular region of interest (10-mm-diameter) measurements[3]. The image quality was determined using the following equation based on references from previous studies [10, 11]: (mean CT value for each nodule—mean CT value for each lung parenchyma) / SD of CT values for each lung parenchyma.

### Nodule detection

**Fig. 4:**
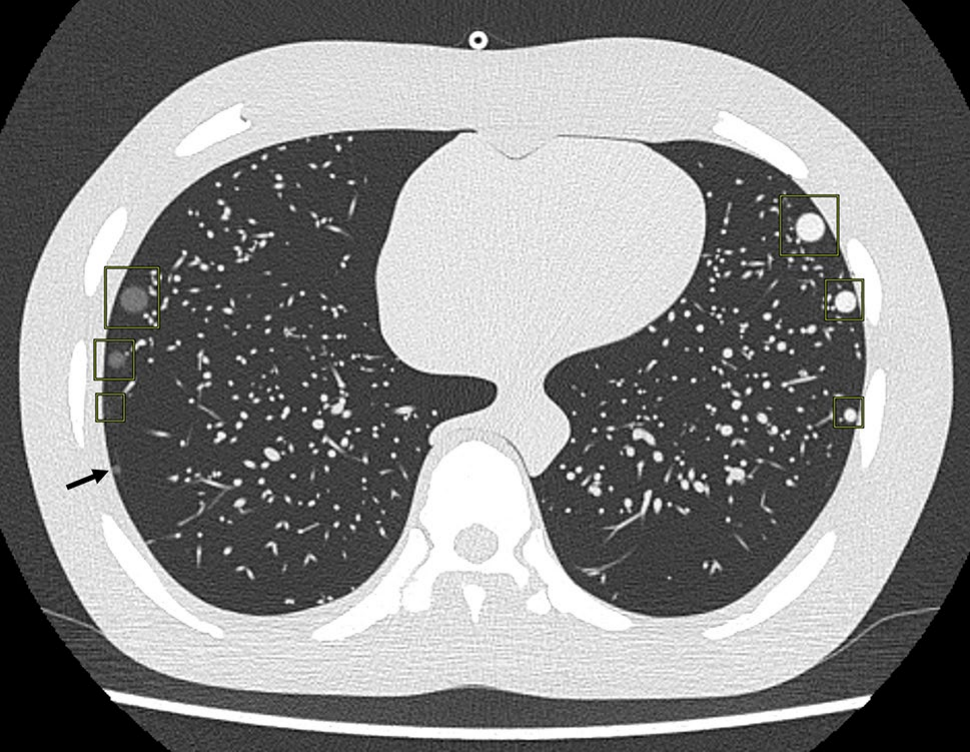
Lung nodules detected by the computer-aided detection (CAD) system. The CAD system detected the location of the artificial lung nodules (rectangles). The 3-mm GGN was not detected by CAD, resulting in a false positive (arrow). The 5-mm GGN and the 3-mm solid nodule were in a craniocaudal displaced position and were not visualized on this slice image

The detection of artificial nodules was performed at the lung window setting (window level, −600 HU; window width, 1500 HU) using a semi-automated method on a CT workstation (Synapse Vincent; Fujifilm Medical, Tokyo). Two radiologists who were aware of the placement locations of the artificial nodules reviewed all images and judged whether each nodule was detectable on axial CT images with a slice thickness of 1 mm. The final judge of the imaging evaluation was determined by consensus of the two radiologists. All images were processed using a commercially available lung nodule CAD system (Lung Nodule Detection Program: 30200BZX00150000 FS-AI688; Fujifilm Medical)[12]. The lung nodule detection algorithm uses a deep learning architecture, and when a chest CT image is input into this architecture, it outputs the location of the lung nodule. The “pulmonary nodule detection function” module was created by having this architecture learn from chest CT images and correct answer data (location information of lung nodules) as learning data. The following CT image settings were the targets for detection by this CAD system: pixel size, 512 × 512; slice thickness, less than 5 mm; spacing between slices, less than 5 mm; target patient, adult; contrast condition, non-contrast; and image reconstruction, lung kernel. The lung nodule settings that were the targets for detection by this CAD system were that the nodule must be located within the lung field and must be sized as follows: (1) solid type, ≥ 3 mm diameter; (2) partially solid type, ≥ 5 mm; and (3) pure GGN type, ≥ 5 mm. The CAD system automatically evaluated axial CT images with a slice thickness of 1 mm and marked the possible location of lung nodules (Fig. 4). Two radiologists counted the number of artificial lung nodules detected by the CAD system and the number of false positives. The causes of false negatives were classified into the following four categories: (1) nodules not targeted by this CAD system; (2) artificial lung nodules located outside the lung field recognized by the CAD system; (3) nodules invisible to the radiologist; and (4) unknown causes.

### Statistical analysis

Statistical analyses were performed by using JMP Pro ver. 17 (SAS Institute Japan, Tokyo). Descriptive statistics were used to describe the detection rate and number of false positives. The nodule detection rate was calculated as a percentage (Number of nodules detected / total number of nodules in combination of each situation which regulated as follows: two types × four sizes × four locations × three repetitions). The nodule detection rate for CAD was calculated in two ways: (1) CAD over all: using all nodules as the denominator (two types × four sizes × four locations × three repetitions = 96), and (2) CAD adaptive: using only nodules of the size defined as the target for CAD detection as the denominator (solid types × four sizes × four locations × three repetitions + GGN types × three sizes × four locations × three repetitions = 84). Differences in detection rate due to different image reconstruction methods were compared using chi-squared tests, and differences in false positives due to different image reconstruction methods were compared using one-way analysis of variance (ANOVA). Statistical significance was accepted at p-values < 0.05.

## Results

### Image quality

Table 1 shows the radiation doses and image quality for the two nodule types using each reconstruction method. The average reconstruction times (maximum dose 120 kV/160 mA, minimum dose 80 kV/10 mA) were FBP (15 s, 15 s), HIR (39 s, 57 s), MBIR (3 min 46 s, 3 min 50 s), and DLR (45 s, 1 min 3 s). HIR, MBIR, and DLR had higher image quality than FBP, and MBIR had the best image quality. The reason for the higher image quality with MBIR was the lower lung CT value due to lower background noise, and the higher SD value of image quality with MBIR was thought to be due to the large image quality value. The diagnostic reference levels for adult chest CT are CTDIvol 13 mGy and DLP 510 mGy・cm, and all of the images taken in this analysis were below these values [13].

**Table 1:** Radiation dose and image quality

Tube voltage		120 kV			80 kV		
Tube current		160 mA	80 mA	10 mA	160 mA	80 mA	10 mA
MAS (mAs)		1240	620	77	1240	620	77
CTDIvol(mGy)		5.1	2.5	0.3	1.2	0.6	0.1
DLP(mGy・cm)		250.9	125.5	15.7	57.7	28.8	3.6
SD Solid nodule	FBP	172 ± 43	223 ± 82	588 ± 258	350 ± 190	481 ± 285	1199 ± 164
	HIR	105 ± 27	110 ± 27	129 ± 17	130 ± 29	141 ± 22	132 ± 32
	MBIR	86 ± 27	88 ± 29	113 ± 19	101 ± 30	110 ± 25	129 ± 29
	DLR	84 ± 32	91 ± 32	114 ± 19	110 ± 31	115 ± 28	125 ± 35
	p-value	< 0.0001	< 0.0001	< 0.0001	< 0.0001	< 0.0001	< 0.0001
Image quality Solid nodule	FBP	11.8 ± 4.1	8.4 ± 2.9	2.7 ± 1.0	5.3 ± 2.2	3.7 ± 1.4	1.0 ± 0.3
	HIR	45.6 ± 6.0	35.6 ± 3.7	24.7 ± 2.2	23.3 ± 4.0	20.7 ± 2.7	30.1 ± 4.9
	MBIR	97.8 ± 17.8	88.5 ± 16.8	65.3 ± 20.8	117.3 ± 44.8	112.7 ± 44	63.8 ± 40.6
	DLR	47.0 ± 5.3	38.4 ± 5.3	26.7 ± 6.0	32.2 ± 2.7	29.4 ± 2.5	25.7 ± 3.7
	p-value	< 0.0001	< 0.0001	< 0.0001	< 0.0001	< 0.0001	< 0.0001
SD GGN	FBP	112 ± 55	149 ± 76	508 ± 12	278 ± 193	391 ± 251	991 ± 205
	HIR	38 ± 7	41 ± 6	58 ± 6	61 ± 9	68 ± 9	59 ± 9
	MBIR	43 ± 5	45 ± 5	55 ± 7	44 ± 8	47 ± 7	58 ± 8
	DLR	40 ± 6	43 ± 5	59 ± 12	54 ± 8	60 ± 12	62 ± 8
	p-value	< 0.0001	< 0.0001	< 0.0001	< 0.0001	< 0.0001	< 0.0001
Image quality GGN	FBP	3.8 ± 1.3	2.7 ± 1.0	0.9 ± 0.3	1.7 ± 0.7	1.2 ± 0.5	0.4 ± 0.4
	HIR	14.7 ± 2.1	11.4 ± 1.3	8.1 ± 0.7	7.6 ± 1.4	6.7 ± 1.0	9.8 ± 1.3
	MBIR	32.1 ± 5.8	29.1 ± 5.5	21.6 ± 6.8	38.6 ± 14.5	37.0 ± 14.5	20.4 ± 13.0
	DLR	15.3 ± 1.8	12.5 ± 1.7	8.9 ± 2.0	10.1 ± 0.9	9.6 ± 0.8	8.7 ± 1.2
	p-value	< 0.0001	< 0.0001	< 0.0001	< 0.0001	< 0.0001	< 0.0001

### Nodule detection rate

**Fig. 5:**
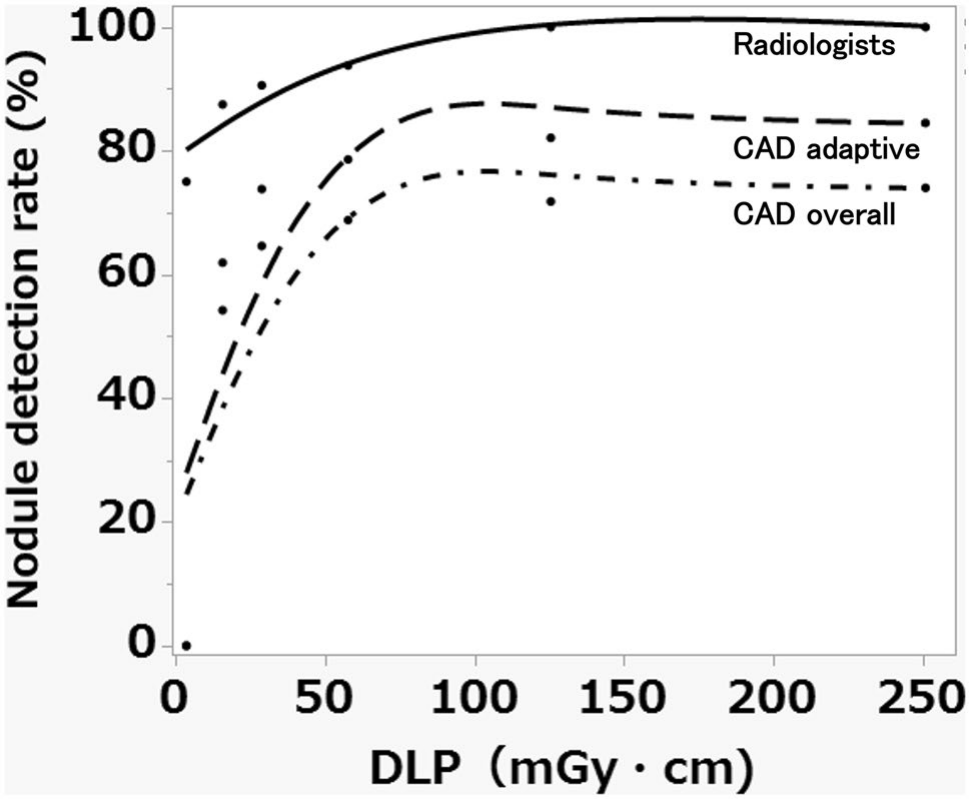
Relationship between the nodule detection rate and radiation dose (dose length product: DLP) in filtered back projection (FBP). FBP showed an exponential increase in the nodule detection rate by both the radiologists and the lung nodule computer-aided detection (CAD) system as the dose increased. CAD adaptive means that the detection rate is calculated based only on the size of the nodules that are the detection targets for CAD (solid type, ≥ 3 mm diameter; pure GGN type, ≥ 5 mm)

Table 2 shows the artificial lung nodule detection rates by scan protocols and reconstruction methods for the radiologists and for the lung nodule CAD. The nodule detection rate of the radiologists was consistently higher than that of CAD across all protocols and reconstruction methods. For both CAD and the radiologists, a trend toward increased nodule detection rates with increasing radiation dose was observed for all image reconstruction methods. In particular, FBP showed an exponential increase in nodule detection rate as the dose increased (Fig. 5). The lowest detection rate was recorded with the lowest radiation dose of 80 kV and 10 mA for all image reconstruction methods. The detection rate by CAD for FBP was 0% regardless of nodule type or size, while that by CAD for DLR was the highest (75.0%).

**Table 2:** Detection rates of artificial nodules by a lung nodule computer-aided detection (CAD) program using various image reconstruction methods and radiation doses

Tube voltage		120 kV			80 kV		
Tube current		160 mA	80 mA	10 mA	160 mA	80 mA	10 mA
FBP	Radiologist	100	100	87.5	93.8	90.6	62.5
	CAD	74.0	71.8	54.2	68.8	64.6	0
	CAD adapt	84.5	82.1	61.9	78.6	73.8	0
HIR (AIDR 3D)	Radiologist	100	100	100	100	100	84.4
	CAD	72.9	75	69.8	72.9	71.9	62.5
	CAD adapt	83.3	85.7	79.8	83.3	82.1	71.4
MBIR (FIRST)	Radiologist	100	100	100	100	100	84.4
	CAD	80.2	78.1	70.8	70.8	66.7	63.5
	CAD adapt	91.7	89.3	81.0	81.0	76.2	72.6
DLR (AiCE)	Radiologist	100	100	100	100	100	87.5
	CAD	74.0	72.9	69.8	71.9	72.9	65.6
	CAD adapt	84.5	83.3	79.8	82.1	83.3	75.0

Each cell is expressed as percentage (number of detected nodule). CAD adapt: CAD adaptive means that the detection rate is calculated based only on the size of the nodules that are the detection targets for CAD (solid type, ≥ 3 mm diameter; pure GGN type, ≥ 5 mm)

*FBP* filtered back projection, *HIR* hybrid iterative reconstruction, *MBIR* model-based iterative reconstruction, *DLR* deep learning reconstruction

Differences in detection rate by CAD depending on nodule type and size are presented in Table 3. The detection rate by CAD of lung nodules was 100% for solid nodules ≥ 5 mm using the DLR method. When using ultra-low-dose imaging at 80 kV and 10 mA, the detection rates of solid nodules ≥ 5 mm and GGNs ≥ 8 mm by CAD were significantly improved with the other three image reconstruction methods (HIR, MBIR, DLR) compared to FBP (p > 0.0001). The detection rates for 3 mm solid nodules and GGNs ≤ 5 mm were lower than those for larger nodules, and no statistically significant differences were observed in the nodule detection rates by CAD due to the different image reconstruction methods. The detection rates for 3 mm GGNs were 0% by all the image reconstruction methods.

**Table 3:** Detection rates by CAD according to nodule type and size

Nodule type		Solid						GGN					
Tube voltages		120 kV			80 kV			120 kV			80 kV		
Tube currents		160 mA	80 mA	10 mA	160 mA	80 mA	10 mA	160 mA	80 mA	10 mA	160 mA	80 mA	10 mA
12 mm	FBP	100	100	91.7^a^	100	100	0.0	100	100	100	100	100	0.0
	HIR	100	100	100	100	100	100	100	100	100	100	100	100
	MBIR	100	100	100	83.3^a^	83.3^a^	100	100	100	100	100	100	100
	DLR	100	100	100	100	100	100	100	100	100	100	100	100
p-value		NA	NA	0.4	0.1	0.1	< 0.0001	NA	NA	NA	NA	NA	< 0.0001
8 mm	FBP	100	100	100	100	100	0.0	100	100	50.0^b^	100	83.3^b^	0.0
	HIR	100	100	100	100	100	100	100	100	100	100	100	100
	MBIR	100	100	100	100	100	100	100	100	100	100	100	100
	DLR	100	100	100	100	100	100	100	100	100	91.7^b^	91.7^b^	100
p-value		NA	NA	NA	NA	NA	< 0.0001	NA	NA	0.0001	0.4	0.3	< 0.0001
5 mm	FBP	100	100	91.7^a^	100	83.3^b^	0.0	41.7	33.3	0.0	16.7	16.7	0.0
	HIR	100	100	100	100	100	75.0^a^	50.0	75.0	25.0	41.7	50.0	0.0
	MBIR	100	100	75.0^a^	83.3^a^	75.0^a^	91.7^a^	66.7	58.3	33.3	33.3	25.0	0.0
	DLR	100	100	100	100	100	100	50.0	41.7	16.7	50.0	50.0	0.0
p-value		NA	NA	0.09	0.1	0.1	< 0.0001	0.7	0.2	0.2	0.4	0.2	NA
3 mm	FBP	50.0	41.7	0.0	33.3	33.3	0.0	0.0	0.0	0.0	0.0	0.0	0.0
	HIR	33.3	25.0	33.3	41.7	25.0	25.0	0.0	0.0	0.0	0.0	0.0	0.0
	MBIR	75.0	66.7	58.3	66.7	50.0	16.7	0.0	0.0	0.0	0.0	0.0	0.0
	DLR	41.7	41.7	41.7	33.3	41.7	25.0	0.0	0.0	0.0	0.0	0.0	0.0
p-value		0.2	0.2	0.02	0.3	0.6	0.3	NA	NA	NA	NA	NA	NA

^a^False negative due to CAD not recognizing lung fields

^b^False negative for unknown reason

*GGN* ground-glass nodule, *FBP* filtered back projection, *HIR* hybrid iterative reconstruction, *MBIR* model-based iterative reconstruction, *DLR* deep learning reconstruction, *NA* not applicable. Probability values were determined by chi-squared test

Differences in the detection rates by CAD depending on nodule type and location are presented in Table 4. In all locations, the nodule detection rate by CAD using FBP was significantly lower than that by CAD using the other image reconstruction methods (HIR, MBIR, DLR) under the ultra-low-dose CT protocol (80 kV/10 mA).

**Table 4:** Detection rates by CAD according to nodule type and location

Nodule type		Solid							GGN					
Tube voltages		120 kV			80 kV				120 kV			80 kV		
Tube currents		160 mA	80 mA	10 mA	160 mA	80 mA	10 mA	160 mA		80 mA	10 mA	160 mA	80 mA	10 mA
Apical Lung	FBP	91.7	83.3	75	75	58.3	0	50		50	25	50	33.3	0
	HIR	91.7	100	100	100	91.7	50	50		75	50	50	50	50
	MBIR	100	100	100	83.3	83.3	75	66.7		58.3	50	50	50	50
	DLR	100	100	91.7	100	100	75	50		50	50	50	50	50
*p*-value		0.5	0.1	0.09	0.1	0.04	0.004	0.8		0.6	0.5	NA	0.7	0.02
Middle Lung	FBP	83.3	75	75	91.7	100	0	75		66.7	50	66.7	66.7	0
	HIR	75	75	83.3	91.7	83.3	100	75		75	75	75	75	50
	MBIR	100	100	100	100	100	91.7	75		75	75	75	75	50
	DLR	75	75	100	75	66.7	100	75		75	66.7	75	75	50
*p*-value		0.3	0.3	0.1	0.2	0.2	< 0.0001	NA		0.9	0.5	0.9	0.9	0.02
Bottom Lung	FBP	100	100	58.3	91.7	75	0	50		50	33.3	50	50	0
	HIR	91.7	75	75	75	75	75	50		50	50	50	50	50
	MBIR	100	91.7	50	58.3	75	66.7	50		50	50	50	75	75
	DLR	91.7	91.7	75	83.3	75	75	50		50	50	50	50	50
*p*-value		0.5	0.2	0.5	0.2	0.5	0.0003	NA		NA	0.7	NA	NA	0.02
Peri- vascular	FBP	75	91.7	75	75	83.3	0	66.7		66.7	41.7	50	50	0
	HIR	75	75	75	75	75	75	75		75	50	66.7	75	50
	MBIR	75	75	83.3	91.7	75	75	75		75	58.3	58.3	50	50
	DLR	75	75	75	75	75	75	75		66.7	50	66.7	66.7	50
*p*-value		NA	0.9	0.9	0.7	0.9	0.0001	0.9		0.9	0.8	0.8	0.5	0.02

Probability values were determined by chi-squared test

*GGN* ground-glass nodule, *FBP* filtered back projection, *HIR* hybrid iterative reconstruction, *MBIR* model-based iterative reconstruction, *DLR* deep learning reconstruction, *NA* not applicable

### False positives and negatives

**Fig. 6:**
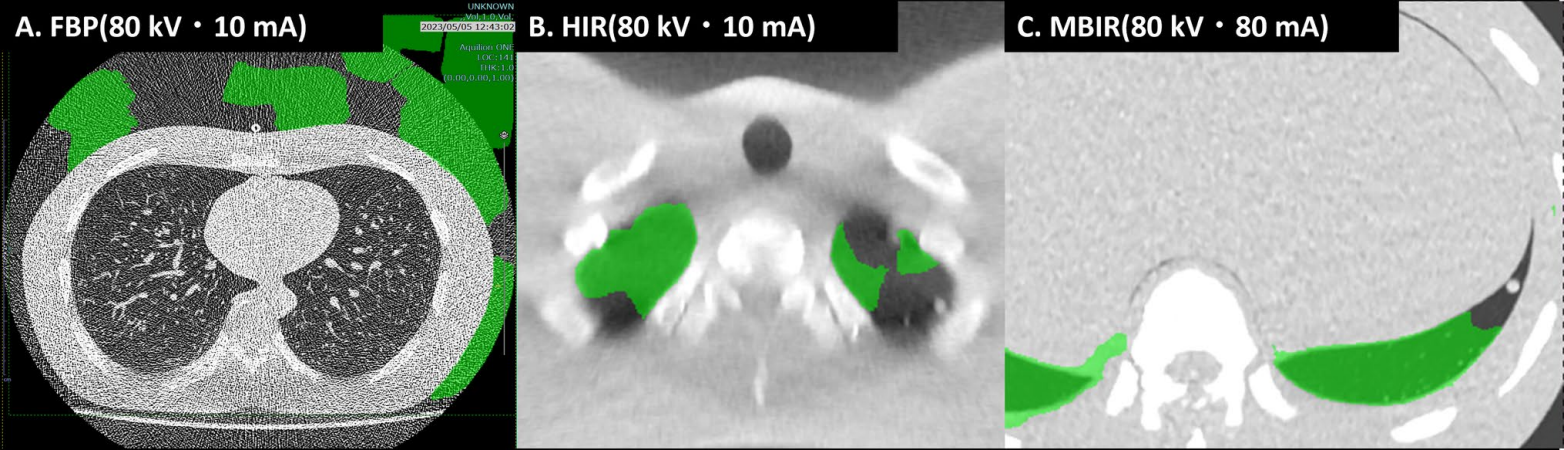
Examples of a false negative involving a lung nodule located outside the CAD-recognized lung field. The green area designates the region identified as a lung field. If a nodule is located in an area recognized as a lung field, it will remain undetected. In A (filtered back projection: FBP; middle lung level, 80 kV, 10 mA), the CAD system fails to recognize the true lung field due to the strong background noise. In B (hybrid iterative reconstruction: HIR; apex lung level, 80 kV, 10 mA) and C (model-based iterative reconstruction: MBIR; basal lung level, 80 kV, 80 mA), the lung regions containing a nodule are not recognized as the lung fields by CAD

False negatives by radiologists, which means nodules are not visible to radiologist, were all included among small-sized nodules (3 mm solid nodules, 3 or 5 mm GGNs). The numbers of false positives by CAD are shown in Table 5. Ultra-low-dose protocols (80 kV/10 mA) showed more false positives (approximately one) for HIR, MBIR, and DLR compared to FBP (zero false positives), and the difference was statistically significant. At higher doses, the different image reconstruction methods showed comparable numbers of false positives (approximately three, respectively). The highest-dose CT protocol (120 kV/160 mA) yielded the greatest number of false positives for FBP (approximately four). The false negatives were attributable to the conditions that 3 mm GGNs were not targeted by this CAD system and/or were not visible to radiologists. The causes of the false negatives for the 3 mm solid and 5 mm GGNs were a mix of nodules invisible to radiologists, nodules located outside the lung field recognized by CAD, and unknown causes. Most of the false negatives for solid nodules were due to the nodules being located outside the lung field recognition by the CAD (Fig. 6). The reason for the false negatives for GGNs ≥ 8 mm was unknown.

**Table 5:** False positives in lung nodule detection programs

Tube voltage	120 kV			80 kV		
Tube current	160 mA	80 mA	10 mA	160 mA	80 mA	10 mA
FBP	4.1 ± 0.4	3.1 ± 0.4	3.3 ± 0.5	3.5 ± 0.4	3.8 ± 0.5	0 ± 0.2
HIR	3.3 ± 0.4	3.3 ± 0.4	2.8 ± 0.5	2.8 ± 0.4	2.8 ± 0.5	0.8 ± 0.2
MBIR	2.8 ± 0.4	2.8 ± 0.4	3.1 ± 0.5	2.9 ± 0.4	3.3 ± 0.5	0.9 ± 0.2
DLR	3.2 ± 0.4	3.3 ± 0.4	3.2 ± 0.5	3.1 ± 0.4	2.8 ± 0.5	1.3 ± 0.2
p-value	0.1	0.7	0.9	0.7	0.3	0.002

*FBP* filtered back projection, *HIR* hybrid iterative reconstruction, *MBIR* model-based iterative reconstruction, *DLR* deep learning reconstruction

## Discussion

This study explored the application of AI-based lung nodule detection programs to ULDCT and demonstrated that the detection performance of this system was influenced by the choice of image reconstruction method, which directly affected image quality. DLR consistently outperformed traditional methods such as FBP, achieving high nodule detection rates even under the lowest dose protocol. However, limitations were observed for small nodules.

Several studies support our findings regarding the superiority of DLR. Mikayama et al. (2022) emphasized that DLR significantly reduced noise and enhanced spatial resolution compared to HIR and MBIR in their phantom study using artificial nodules [3]. Similarly, Cui et al. (2022) demonstrated that AI-based detection systems combined with advanced image reconstruction can achieve sensitivity levels surpassing those of radiologist double readings in low-dose CT protocols [8]. Yao et al. (2022) demonstrated that DLR significantly enhances the signal-to-noise ratio (SNR) and contrast-to-noise ratio (CNR), which are critical for nodule visibility under reduced radiation doses [4]. Similarly, Franck et al. (2021) noted that DLR preserves image fidelity and minimizes artificial “plastic-like” textures commonly associated with iterative reconstruction methods [5]. Our study found that DLR-reconstructed images in lowest dose CT achieved the highest nodule detection rates for both radiologists and CAD system. Although the nodule detection rate by DLR was clearly superior to that by FBP, the difference was minimal when compared to the superiority of DLR over HIR and MBIR. This is likely attributable to the fact that the image quality showed little variation among reconstruction methods other than FBP. Another possible reason for this result may be that the training data for CAD development incorporates images generated using various reconstruction methods from multiple vendors and is designed to ensure robust CAD performance as long as a minimum standard of image quality is maintained.

This study looks more closely at the reasons for the false negatives in CAD systems, providing a new perspective that has not been highlighted before. In the CAD system utilized in this study, the program initially identifies the lung field and subsequently detects potential lung nodules within it. As a result, lung nodules situated in regions that the program classifies as being outside the lung field remain undetected. Consequently, in the FBP reconstruction of ULDCT scans with substantial noise, CAD was unable to accurately identify the lung fields, leading to false negatives. In order to ensure that the CAD system could detect nodules, it was necessary to reduce noise to a level at which the lung fields can be reliably identified. The present analysis showed that advanced image reconstruction technologies such as HIR, MBIR, and DLR can be applied to sufficiently reduce the noise in ULDCT images (80 kV/10 mA) and ensure the proper functionality of CAD [12].

The integration of the advanced image reconstruction technologies such as HIR, MBIR, and DLR with AI-based CAD systems holds promise for enhancing lung cancer screening. In this study, the 80 kV/160 mA protocol was close to the average dose for low-dose lung cancer screening, and with this protocol, CAD sensitivity was greater than the official 78.4% [12] for all reconstruction methods, including FBP. Both 120 kV/10 mA and 80 kV/10 mA are classified as ultra-low-dose CT protocols, with 80 kV/10 mA being the lowest dose protocol currently configurable on available devices. It was suggested that the sensitivity of CAD could be ensured by using the advanced reconstruction method with the 120 kV/10 mA protocol; however, the sensitivity was not satisfactory with the 80 kV/10 mA protocol. By enabling accurate nodule detection at significantly lower radiation doses, this approach aligns with the ALARA (As Low As Reasonably Achievable) principle, which is critical for minimizing cumulative radiation exposure in high-risk populations undergoing repeated screenings[4, 5, 7, 14, 15]. Additionally, the improved detection rates for small nodules suggest potential benefits for early-stage lung cancer diagnosis, which is pivotal for improving patient outcomes. Our findings expand on previous literature by highlighting the differential impact of reconstruction methods on CAD performance. While Mikayama et al. and Cui et al. emphasized the benefits of DLR in radiologist-assisted detection, our study demonstrates the efficacy of DLR for AI-driven detection, bridging a critical gap in the existing body of knowledge [3, 4, 8]. Moreover, the observed sensitivity variations across nodule sizes and locations underscore the need for tailored reconstruction protocols to optimize detection accuracy.

Several study limitations bear mention. First, this study employed a chest phantom, which, while valuable for controlled experimentation, cannot fully replicate the complexity of factors affecting in vivo conditions, such as motion artifacts and patient variability. Second, this study did not take into account laterality in nodule detection rates by the CAD system. Third, this study did not consider the ratio of imaging devices and reconstruction techniques included in the training data for the CAD system. Finally, the evaluation was restricted to a single AI-based CAD system, limiting the generalizability of findings across different vendors and algorithms. Future research should explore multi-center, multi-vendor studies to validate these results and expand their applicability.

In conclusion, this study underscores the pivotal role of image reconstruction techniques in enhancing the efficacy of AI-based nodule detection programs in ULDCT. Nonetheless, additional research will be needed to address the challenges of small nodule detection and validation with clinical data before widespread clinical adoption.
